# Supplementary material for: Identification of potential aggregation hotspots on Aβ42 fibrils blocked by the anti-amyloid chaperone-like BRICHOS domain
Source: Nat Commun. 2024 Feb 1;15:965. doi: 10.1038/s41467-024-45192-4 (PMC10834949; doi:10.1038/s41467-024-45192-4)
Supplement: Supplementary file 1 — Supplementary Information [file 41467_2024_45192_MOESM1_ESM.pdf]

Supplementary Information for

Identification of potential aggregation hotspots on Aβ42 fibrils blocked by the anti-amyloid chaperone-like BRICHOS domain

Rakesh Kumar[1], Tanguy Le Marchand[2], Laurène Adam[1], Raitis Bobrovs[3], Gefei Chen[1], Jēkabs Fridmanis[3], Nina Kronqvist[1], Henrik Biverstål[1], Kristaps Jaudzems[3], Jan Johansson[1], Guido Pintacuda[2], Axel Abelein[1,*]

[1] Department of Biosciences and Nutrition, Karolinska Institutet, 141 83 Huddinge, Sweden

[2] Université de Lyon, Centre de Resonance Magnétique Nucléaire (CRMN) à Très Hauts Champs de Lyon (UMR 5082 - CNRS, ENS Lyon, UCB Lyon 1), 69100 Villeurbanne, France

[3] Department of Physical Organic Chemistry, Latvian Institute of Organic Synthesis, Riga, Latvia

*corresponding author: Axel Abelein

Email: axel.abelein@ki.se

Supplementary Information Figures

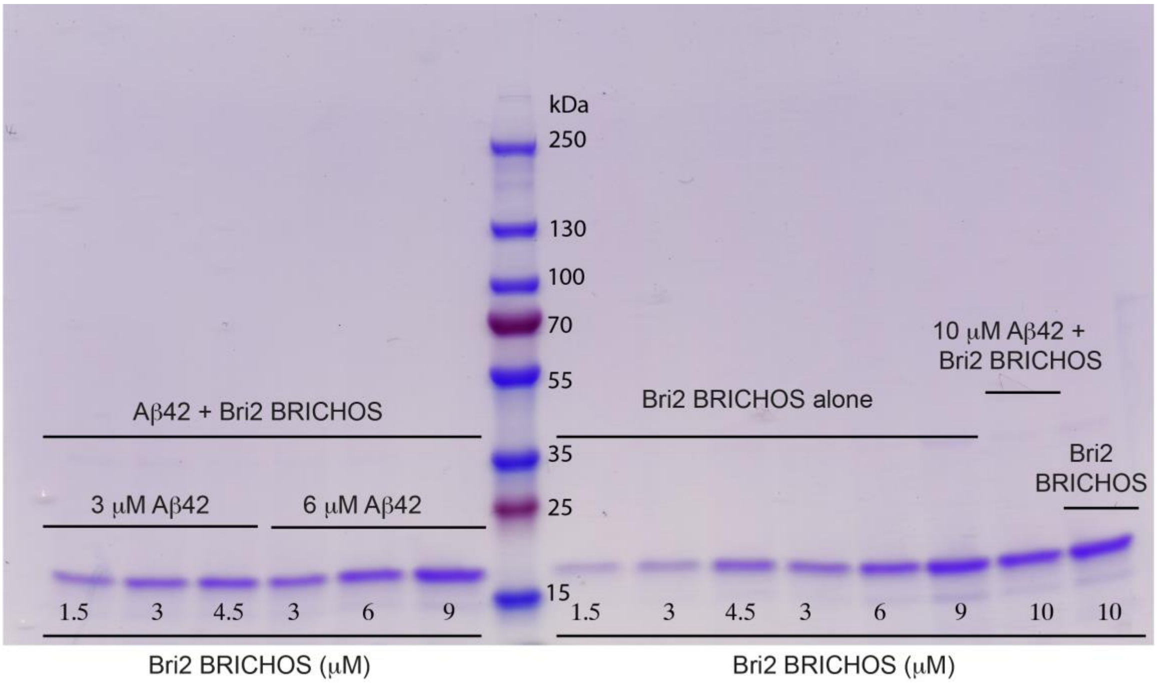

Supplementary Figure 1. SDS-PAGE analysis of BRICHOS binding to Aβ42 fibrils – uncropped SDS-PAGE gel of Figure 1C. Different concentrations of BRICHOS (1.5 to 10 μM) were co-incubated with different concentrations of Aβ42 (3, 6 and 10 μM) to form fibrils. The samples were centrifuged and the supernatant loaded on the SDS-PAGE gel. The results show that most BRICHOS is still in the supernatant and not bound to the fibrils. The last two lanes are shown in Figure 1C.

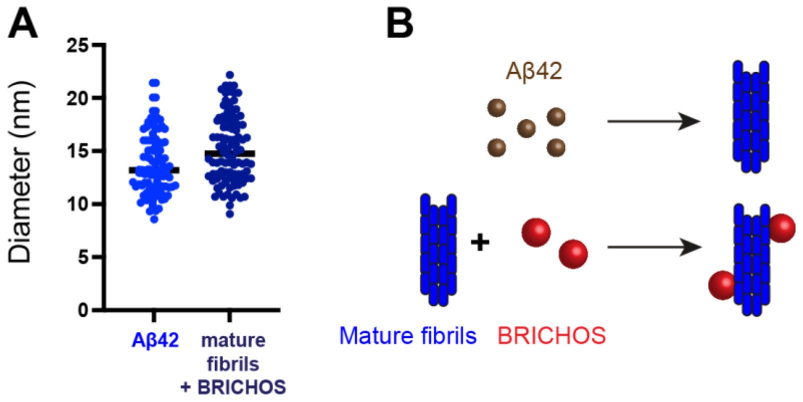

Supplementary Figure 2. Fibril diameter of BRICHOS added to mature Aβ42 fibrils. (A) Analysis of fibril diameters of mature Aβ fibrils with and without added BRICHOS reveals very similar values. n=100 independent measurements are shown where the line corresponds to the mean. Source data are provided as Source Data file. **(B)** Schematic overview about sample preparation.

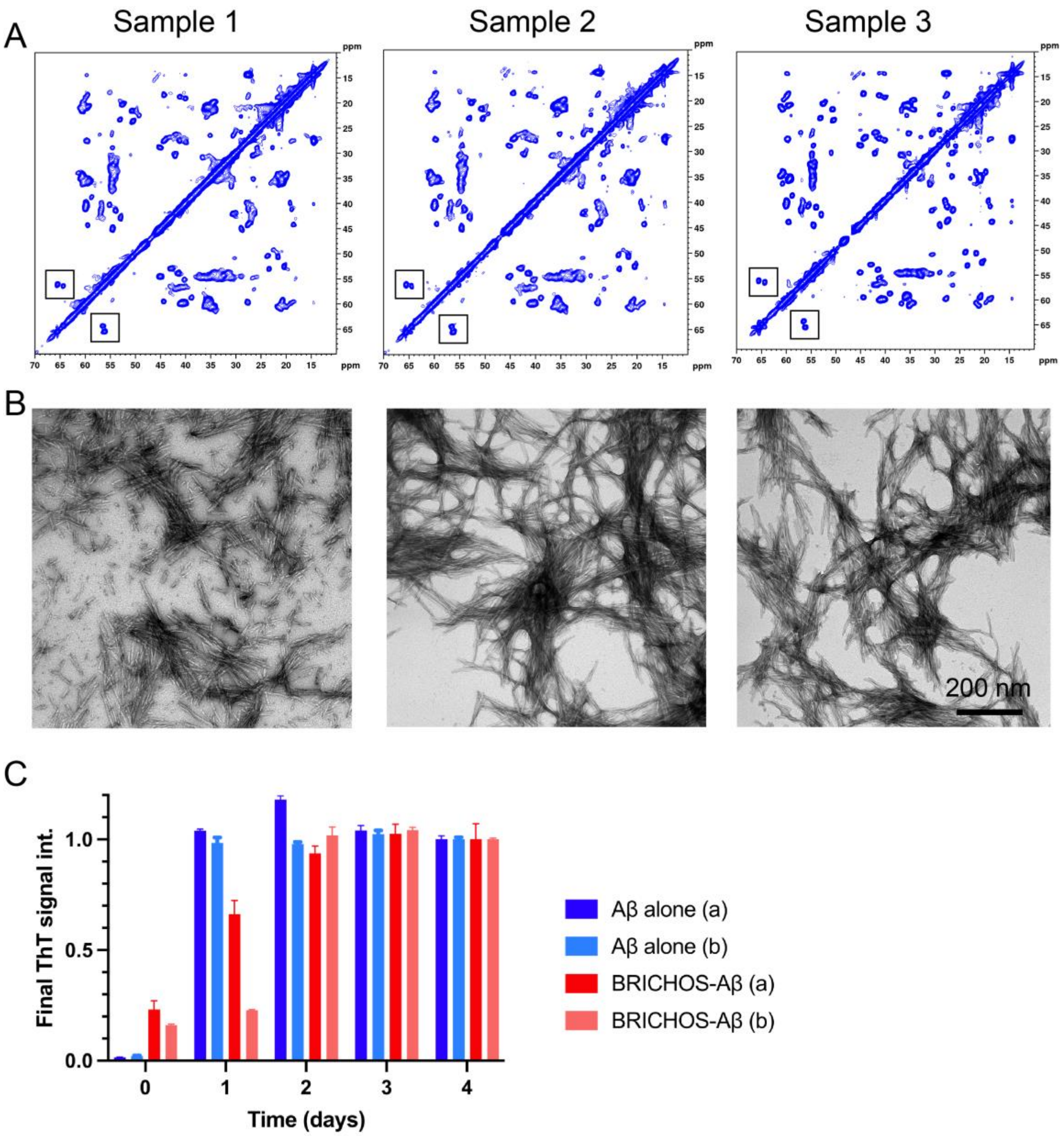

Supplementary Figure 3. ^{13}C-^{13}C DARR spectra, EM images of different sample preparations for Aβ42 alone fibrils and final ThT signal intensity. (A) ^{13}C-^{13}C DARR spectra and **(B)** EM images of different sample preparations as described in Supplementary Table 2. Sample 3 exhibits the sharpest signals and the two distinct peaks at around 55-60 ppm correspond to the two serine signals (marked in the box), indicating one major fibril morphology. **(C)** Final ThT intensities of Aβ42 alone and BRICHOS-Aβ42 co-incubated samples at different time points. The samples were incubated in the same manner as used for the preparation of the ssNMR samples, where small aliquots were taken and the ThT intensity was measured in a ThT plate using four replicates. The individual errors represent the standard derivations of these four replicates and the experiment was repeated twice, referred to as (a) and (b).

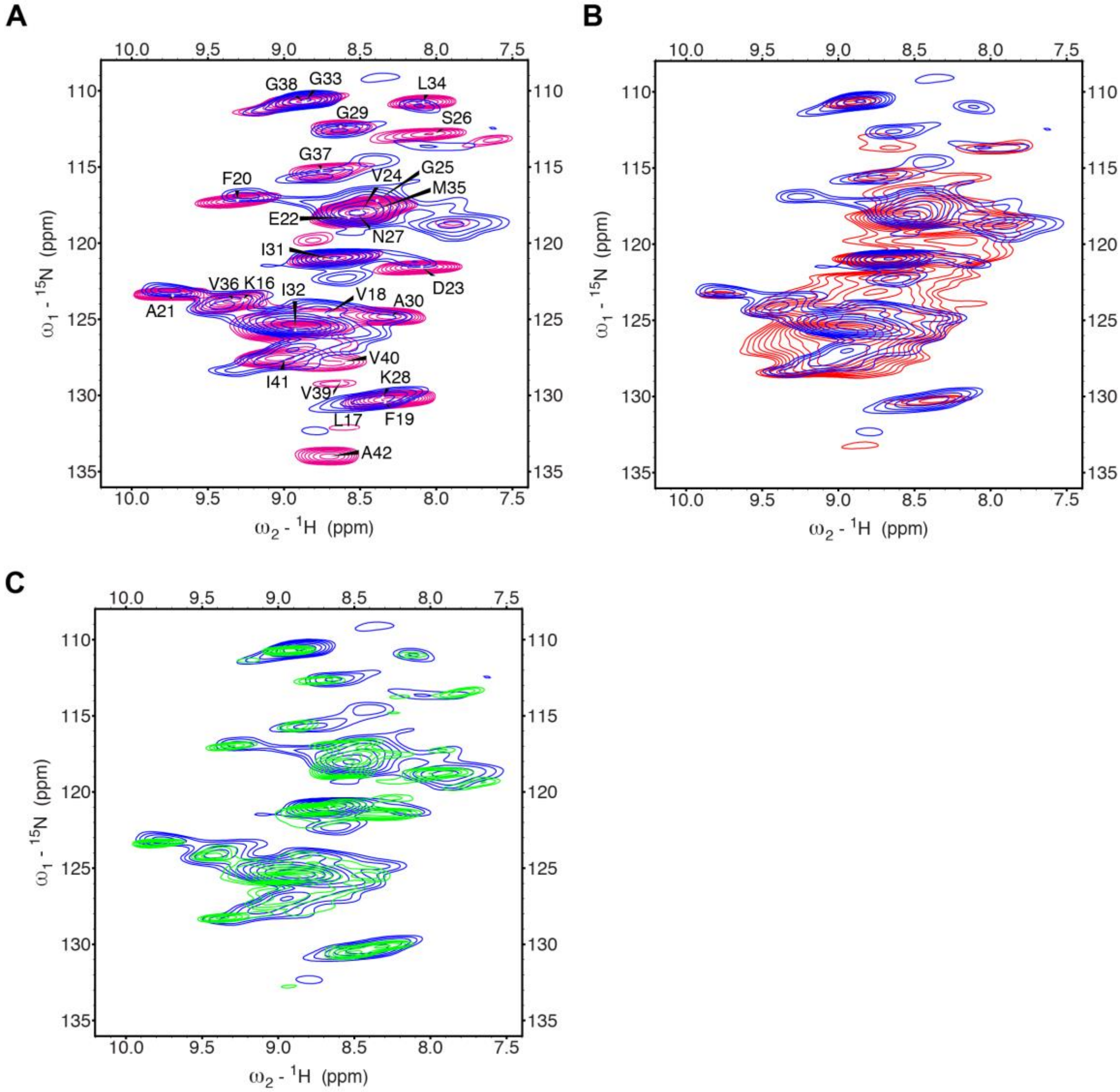

Supplementary Figure 4. ^{1}H,^{15}N-correlation spectra of Aβ42 fibrils and the effect of BRICHOS. (A) ^{1}H,^{15}N-correlation spectra of Aβ42 fibrils alone (blue) overlaid with the spectrum and assignment as published by Griffin & coworkers (pink) [1]. **(B,C)** Overlap of ^{1}H,^{15}N-correlation spectra of Aβ42 fibrils alone (blue) with (B) BRICHOS-Aβ42 co-incubated fibrils (red) and (C) BRICHOS added to mature Aβ42 fibrils (green).

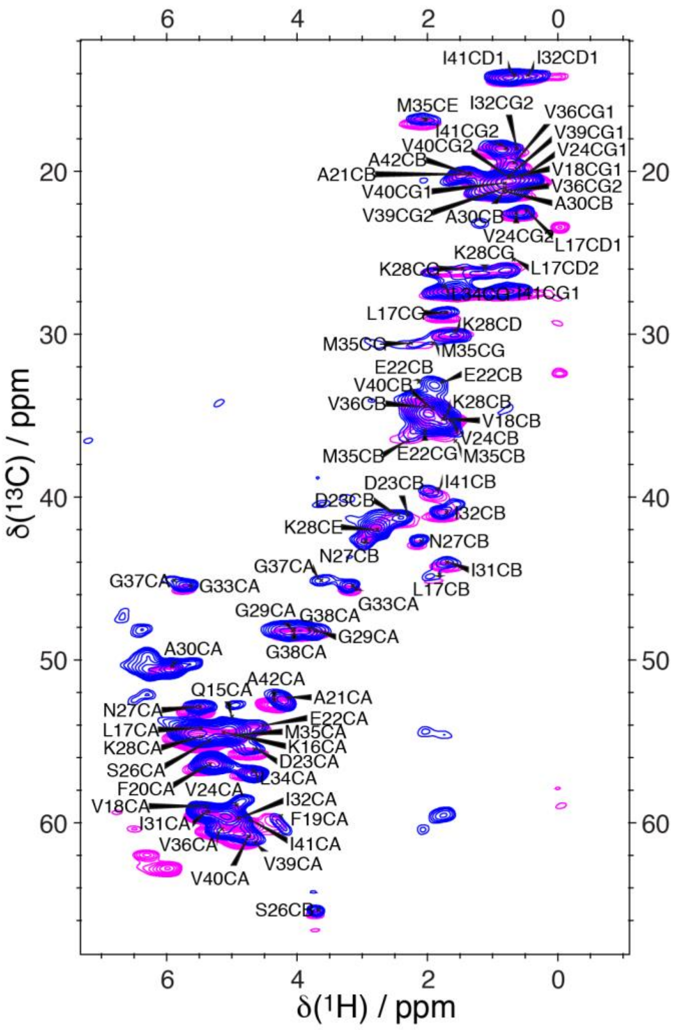

Supplementary Figure 5. ^{1}H,^{13}C-correlation spectra of Aβ42 fibrils. Comparison of Aβ42 fibrils recorded here (blue) with the published spectrum and assignment of Griffin & coworkers (pink) [1].

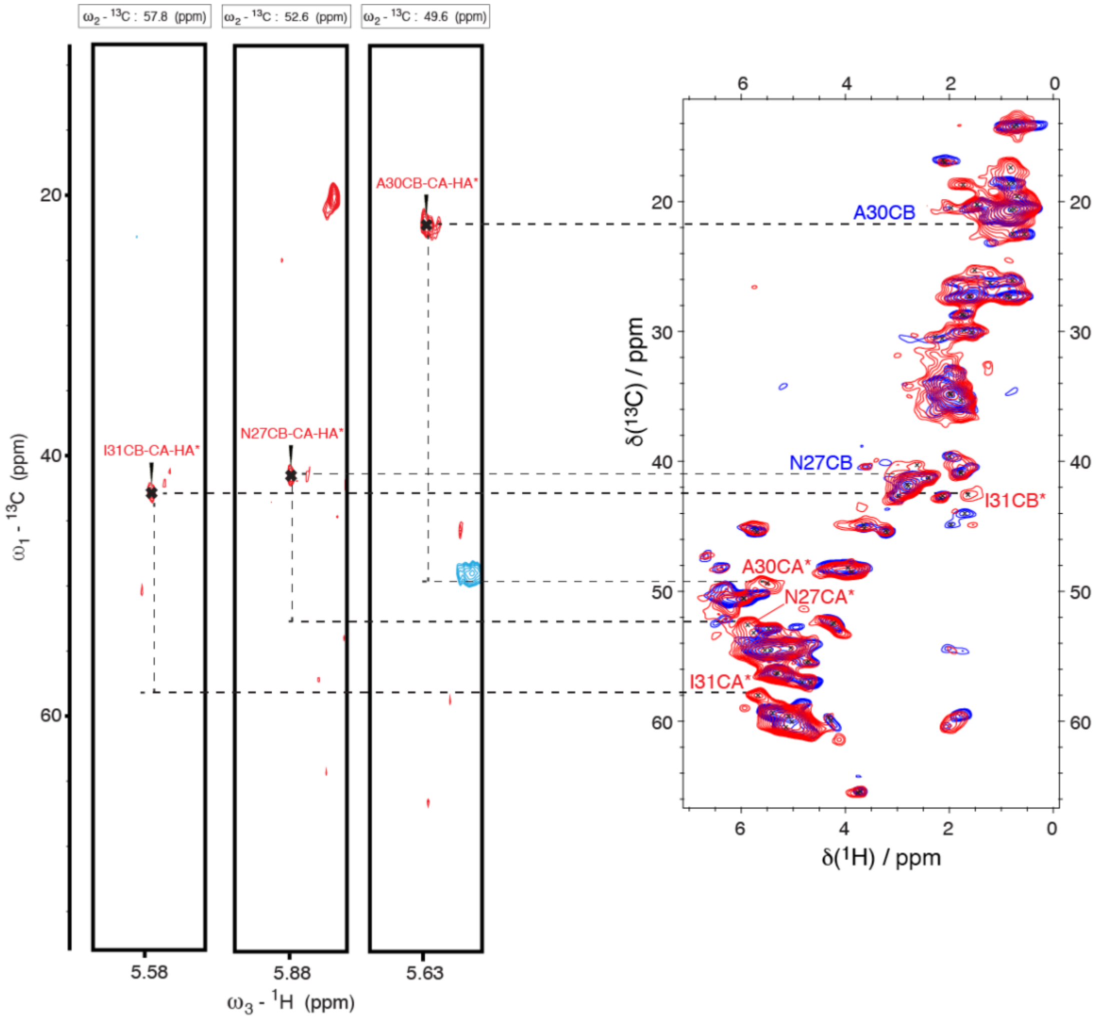

Supplementary Figure 6. Assignment of shifted peaks of BRICHOS-Aβ42 fibrils using (H)CBCAH experiment. Spectra are shown for Aβ42 fibrils alone (blue) and BRICHOS-Aβ42 fibrils (red). Strips of (H)CBCAH experiment of the three doubled peaks, marked with a star, in the CA-region using previous assignment of Aβ42 alone fibrils [1].

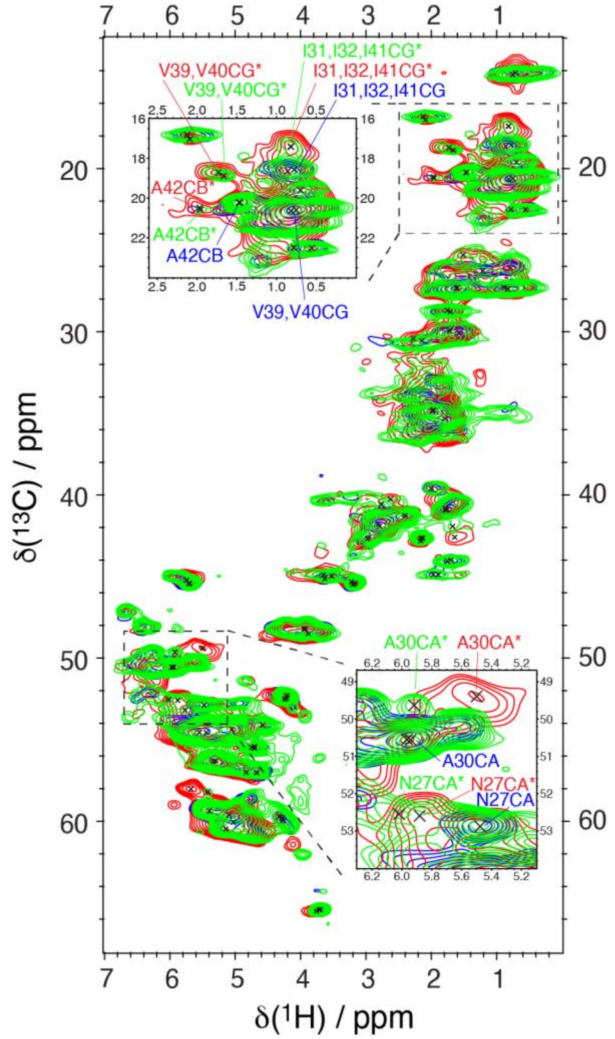

Supplementary Figure 7. ^{1}H,^{13}C-correlation spectra. The ^{1}H,^{13}C-correlation spectra of Aβ42 fibrils alone (blue), BRICHOS added to mature Aβ42 fibrils (green) and BRICHOS-Aβ42 co-incubated samples (red) are shown. The inserts represent different zoomed regions. The assignments of the doubled peaks visible in the BRICHOS samples are marked with a star (*).

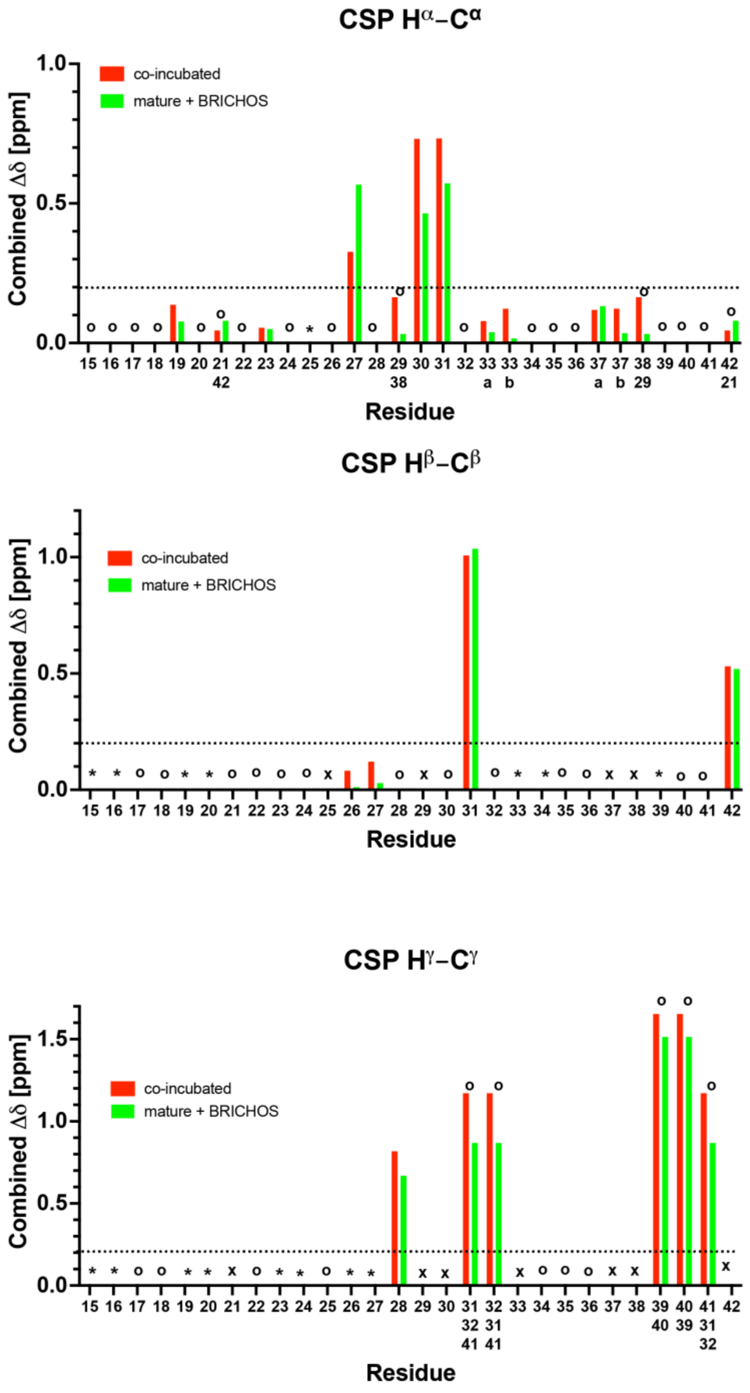

Supplementary Figure 8. Chemical shift differences between doubled peaks in ^{1}H,^{13}C-correlation spectra. Combined (^{1}H and ^{13}C) chemical shift changes between the set of signals of mature Aβ42 fibrils alone and the new set of peaks visible in co-incubated BRICHOS-Aβ42 fibrils (red) and those visible in BRICHOS added to mature Aβ42 fibrils (green). Due to the low intensity of the doubled peaks of the BRICHOS added to mature Aβ42 fibrils samples, these peaks were assigned based on the doubled peaks of the co-incubated BRICHOS-Aβ42 fibril sample. Missing assignments are labeled with an asterisk (*), crosses (x) refer to nuclei that are not present in the corresponding residue and overlaps are marked by circles (o) where ambiguous assignments for the residues are stated.

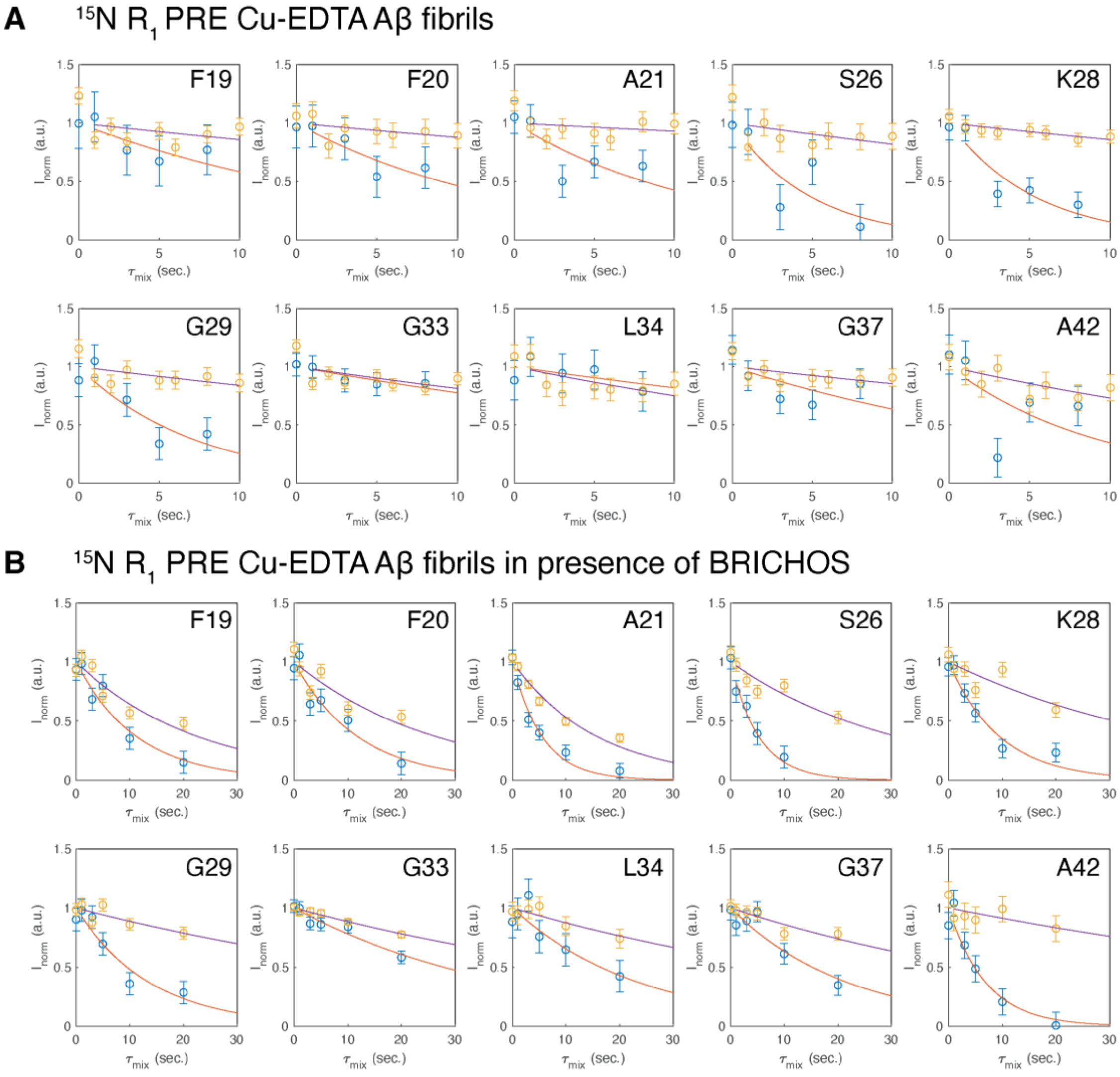

Supplementary Figure 9. PRE effect of Cu-EDTA. Longitudinal ^{15}N R_1 relaxation rates and exponential fits for assigned residues in ^{1}H,^{15}N-correlation spectra of Aβ42 fibrils alone (A) and with BRICHOS added to mature Aβ42 fibrils (B), before (orange) and after addition (blue) of Cu-EDTA to determine the PRE effect of Cu-EDTA [2, 3, 4].

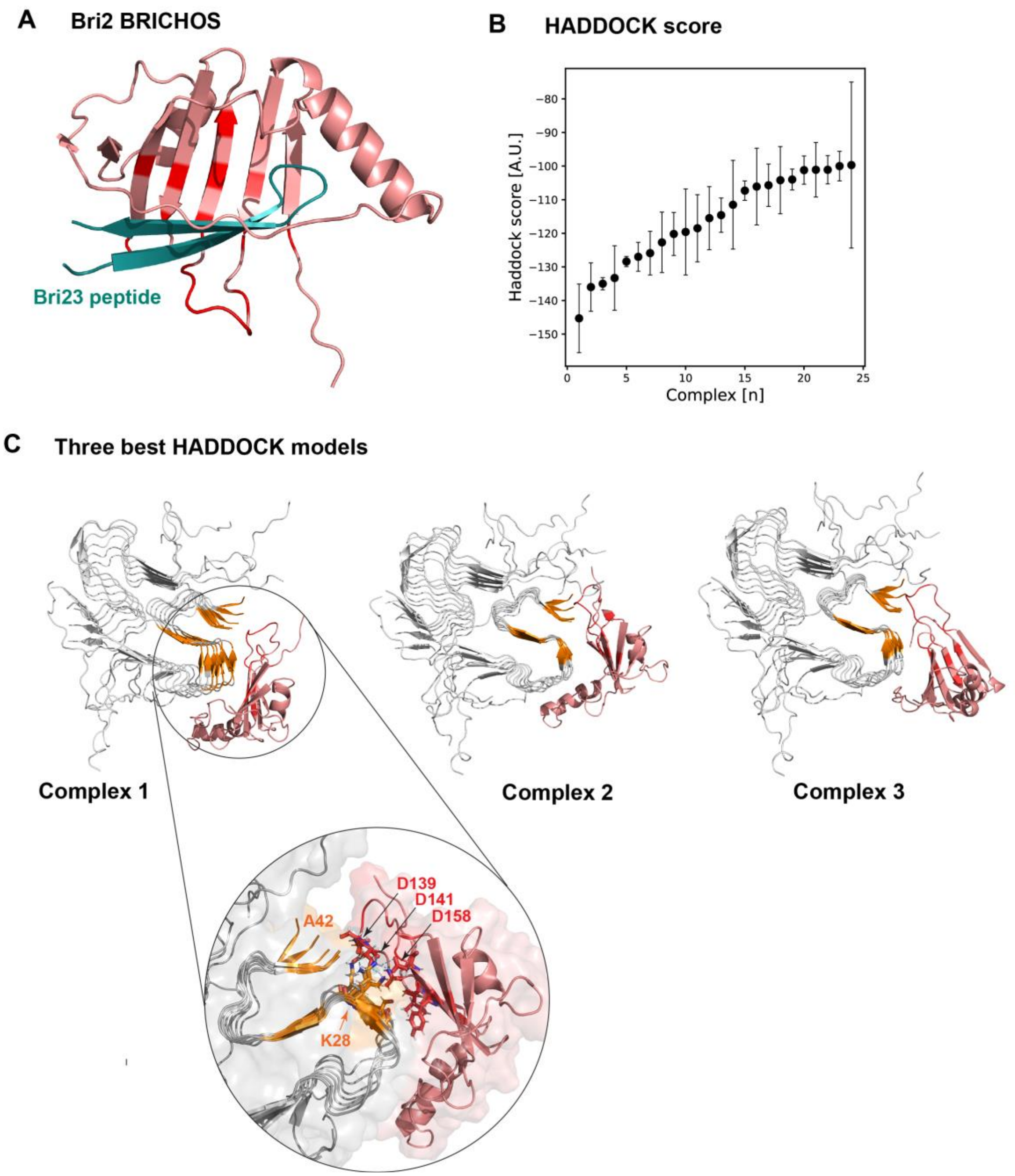

Supplementary Figure 10. HADDOCK models of BRICHOS-Aβ42 complexes. (A) AlphaFold2 model of R221E Bri2 BRICHOS aligned with the Alpha2 model of full-length Bri2 protein where the natural amyloidogenic client, the Bri23 peptide, is shown. Residues close (within 5 Å) to Bri23 peptide are marked in red. **(B)** Haddock score of modeled complexes is presented. **(C)** The three best Haddock models are visualized where the interacting residues on Aβ42 fibril are marked in orange and on BRICHOS in red. The zoom-in graph shows the ionic interaction network between K28 on the Aβ42 fibril surface and D139, D141 and D158 of BRICHOS. MD models and the best HADDOCK complexes are provided as Supplementary data 1.

Supplementary Information Tables

Supplementary Table 1. Kinetic rate constants and apparent binding constants determined by SPR for BRICHOS binding to Aβ42 fibrils

$k_{on,1}$ $[M^{-1}\ s^{-1}]$	$k_{off,1}$ $[s^{-1}]$	$k_{on,2}$ $[M^{-1}\ s^{-1}]$	$k_{off,2}$ $[s^{-1}]$	K_{D1} $[\mu M]$	K_{D1} $[nM]$
$(3.61 \pm 0.03) \cdot \mathbf{10^2}$	$(6.69 \pm 0.04) \cdot 10^{-3}$	$(1.36 \pm 0.01) \cdot 10^4$	$(1.76 \pm 0.01) \cdot 10^{-4}$	18.5 ± 0.2	12.9 ± 0.2

Supplementary Table 2. Conditions used for preparation of Aβ fibril for ssNMR studies in the literature.

Reference	Seeding	Aβ	[Aβ]	Shaking	Temp.	Buffer	Additives
Wälti et al., PNAS, 2016 [5]	10% for 3 generations	rec. 1-42	30 µM (or 100 µM)	350 rpm	37 °C	100mM NaP, pH 7.4	100 mM NaCl, 100 µM ZnCl
Xiao et al., Nat Struc Mol Biol, 2015 [6]	5% for > 4 generations	syn. 1-42	50 µM	slowly agitated for 3-4d	RT	10mM NaP, pH 7.4	No
Colvin et al., JACS, 2016 [7]	no, simply incubated in Falcon tubes	rec. M1-42	10-50 µM	no	RT	20mM NaP, pH 8, 0.2mM EDTA, 0.02% NaN3	No

Supplementary Table 3. Screening conditions for homogeneous production of Aβ42 fibrils alone.

Samples	Seeding	Aβ	[Aβ]	Shaking	Temp.	Buffer	Additives
Sample 1	10% for 1 generations	rec. 1-42	30 µM	no	25 °C	20mM NaP, pH 8, 0.02% NaN3, 0.2mM EDTA,	No
Sample 2	10% for 4 generations	rec. 1-42	30 µM	no	25 °C	20mM NaP, pH 8, 0.02% NaN3, 0.2mM EDTA,	No
Sample 3	10% for 4 generations	rec. 1-42	30 µM	300-350 rpm	25 °C	20mM NaP, pH 8, 0.02% NaN3, 0.2mM EDTA,	No

Supplementary Table 4. Transverse relaxation time T_2 in ms of different fibril samples.

Nucleus	Griffin et al.	Aβ42 alone	BRICHOS-Aβ42
^{1}H	2.26	2.25	2.26
^{15}N	68	66	49

Supplementary References

1. Bahri S*, et al.* (1)H detection and dynamic nuclear polarization-enhanced NMR of Abeta1-42 fibrils. *Proc Natl Acad Sci U S A* **119**, (2022).

2. Öster C*, et al.* Characterization of Protein-Protein Interfaces in Large Complexes by Solid-State NMR Solvent Paramagnetic Relaxation Enhancements. *J Am Chem Soc* **139**, 12165-12174 (2017).

3. Aucoin D*, et al.* Protein-solvent interfaces in human Y145Stop prion protein amyloid fibrils probed by paramagnetic solid-state NMR spectroscopy. *J Struct Biol* **206**, 36-42 (2019).

4. Wickramasinghe NP*, et al.* Nanomole-scale protein solid-state NMR by breaking intrinsic 1HT1 boundaries. *Nat Methods* **6**, 215-218 (2009).

5. Wälti MA*, et al.* Atomic-resolution structure of a disease-relevant Abeta(1-42) amyloid fibril. *Proc Natl Acad Sci U S A* **113**, E4976-4984 (2016).

6. Xiao Y*, et al.* Abeta(1-42) fibril structure illuminates self-recognition and replication of amyloid in Alzheimer's disease. *Nat Struct Mol Biol* **22**, 499-505 (2015).

7. Colvin MT*, et al.* Atomic Resolution Structure of Monomorphic Abeta42 Amyloid Fibrils. *J Am Chem Soc* **138**, 9663-9674 (2016).
