# Supplementary material for: Identification of potential aggregation hotspots on Aβ42 fibrils blocked by the anti-amyloid chaperone-like BRICHOS domain
Source: Nat Commun. 2024 Feb 1;15:965. doi: 10.1038/s41467-024-45192-4 (PMC10834949; doi:10.1038/s41467-024-45192-4)
Supplement: Supplementary file 3 — Description of additional supplementary files [file 41467_2024_45192_MOESM3_ESM.pdf]

Description of additional supplementary files

Supplementary Data 1: Folder "ab42": The pdb structures of five randomly selected frames from the MD simulation of the Aβ42 fibril structure based PDB structure 5KK3 [https://doi.org/10.2210/pdb5KK3/pdb], where the 10 missing N-terminal amino acids were added, are provided.
Folder "bri2": The pdb structures of five randomly selected frames from the MD simulation of the R221E Bri2 BRICHOS structure, based on the AlphaFold2 model, are provided.
Folder "complex": The pdb structures of the three best HADDOCK models of the BRICHOS-Aβ42 fibril complex are provided.
